# The Prognostic Nutritional Index May Predict Left Atrial Appendage Thrombus or Dense Spontaneous Echo Contrast in Patients With Atrial Fibrillation

**DOI:** 10.3389/fcvm.2022.860624

**Published:** 2022-04-29

**Authors:** Zhao Wang, Binhao Wang, Guohua Fu, Bin He, Huimin Chu, Shengmin Zhang

**Affiliations:** ^1^Department of Ultrasonography, Ningbo First Hospital, Ningbo, China; ^2^Arrhythmia Center, Ningbo First Hospital, Ningbo, China

**Keywords:** prognostic nutritional index, left atrial appendage thrombus, spontaneous echo contrast, non-valvular atrial fibrillation, immunonutritional status

## Abstract

**Objectives:**

The prognostic nutritional index (PNI) is an independent predictor of adverse outcomes in patients with cardiovascular diseases. The presence of left atrial appendage thrombus (LAAT) or spontaneous echo contrast (SEC) is associated with ischemic stroke. The present study aimed to investigate the relationship between the PNI and LAAT/dense SEC in patients with non-valvular atrial fibrillation (AF).

**Methods:**

In patients with non-valvular AF, we compared demographics, clinical characteristics, and prevalence of LAAT/dense SEC according to the levels of the PNI. The relationship between the PNI and LAAT/dense SEC was observed.

**Results:**

A total of 406 patients with non-valvular AF were consecutively included from March 2015 to February 2019. Of the study population, 53 patients had LAAT/dense SEC. The percentages of LAAT/dense SEC were 20.4, 14.1, and 4.5% in subjects from the lowest to the highest tertile of the PNI, respectively. Multivariate logistic analysis demonstrated that the PNI was an independent predictor for LAAT/dense SEC (OR 0.89; 95% CI, 0.82–0.97; *P* = 0.007). Receiver operating characteristic curve analysis revealed that the optimal cutoff value of the PNI for predicting LAAT/dense SEC was 48.0 (area under the curve: 0.68; 95% CI, 0.61–0.75; *P* < 0.001). The sensitivity and specificity were 83.0 and 47.6%, respectively. The risk of LAAT/dense SEC in patients with a PNI ≤ 48.0 was 2.57-fold higher than that in those with a PNI > 48.0.

**Conclusion:**

The PNI, calculated based on serum albumin and lymphocyte count, was inversely correlated with LAAT/dense SEC in patients with non-valvular AF. Therefore, it may be considered a predictor for LAAT/dense SEC.

## Introduction

Atrial fibrillation (AF) is the most sustained cardiac arrhythmia in the clinical setting (1). Ischemic stroke is one of the most severe complications caused by AF (2). Approximately 90% of thrombi originate in the left atrial appendage (LAA) (3). Spontaneous echo contrast (SEC) is a marker for a hypercoagulable state (4). Previous investigations indicated that dense SEC was associated with ischemic stroke even after catheter ablation (5) and LAA closure (6). Transesophageal echocardiography is usually performed in patients with AF who were candidates for procedure or cardioversion to exclude thrombi in the left atrium (LA). However, it was not a routine examination for the other AF patients during outpatient follow-up. Therefore, it is essential to identify patients at risk of LAA thrombus (LAAT) or SEC and provide appropriate treatment to protect patients from stroke.

The prognostic nutritional index (PNI) is a simple index comprised of serum albumin and lymphocyte count that reflects the immunonutritional status. It was shown to be associated with poor outcomes of cardiovascular diseases (7–9). Recently, a low PNI was reported as an independent predictor for incident AF (10) and AF recurrence after ablation (11). However, whether PNI could serve as a prognostic biomarker for LAAT/dense SEC remains unknown. Thus, we conducted this study to assess the association between PNI and LAAT/dense SEC in patients with AF.

## Materials and Methods

### Study Population

**FIGURE 1:**
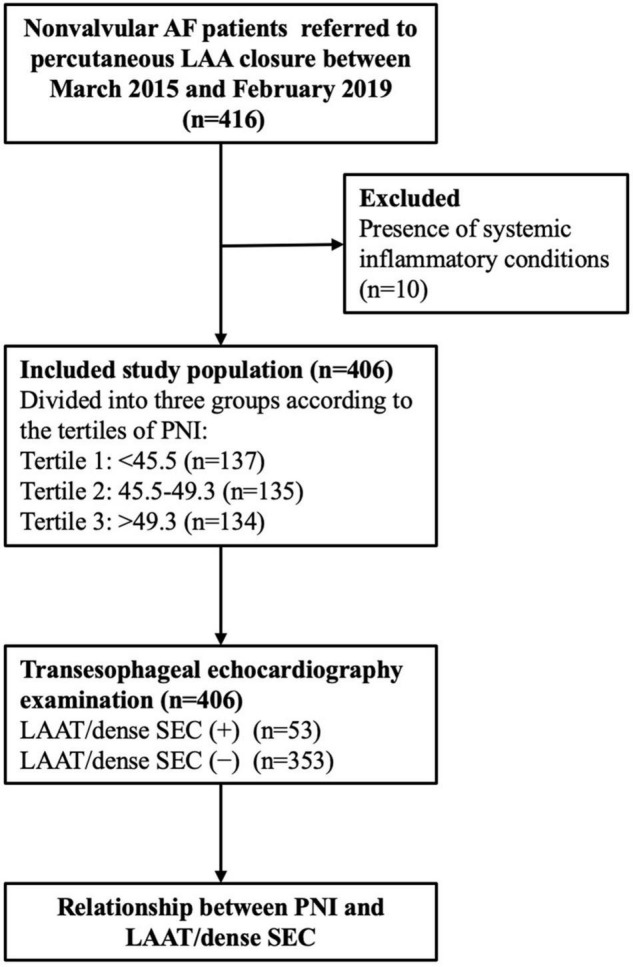
Study flow chart. AF, atrial fibrillation; LAA, left atrial appendage; PNI, prognostic nutritional index; LAAT, left atrial appendage thrombus; SEC, spontaneous echo contrast.

Non-valvular AF patients who were candidates for percutaneous LAA closure between March 2015 and February 2019 were reviewed. The exclusion criteria were patients with (1) mechanical valves or moderate-to-severe mitral stenosis; (2) chronic kidney disease (estimated glomerular filtration rate < 60 mL/min/1.73 m^2^), malignancy, connective tissue diseases, or chronic systemic disease; and (3) recent acute infectious or inflammatory disease, high body temperature > 38°C, and white blood cell count > 12 × 10^9^/L or < 4 × 10^9^/L. Finally, a total of 406 patients were included in the study (Figure 1).

This study was conducted in compliance with the law protecting personal data in accordance with the guidelines of the Helsinki Declaration. The study was approved by the Ethics Committee of Ningbo First Hospital and written informed consent was obtained from all patients.

### Data Collection

The demographic and clinical data were collected from the electronic medical database. Oral anticoagulation (OAC) medication, including warfarin, dabigatran, and rivaroxaban, was also recorded. Blood samples were collected for analysis on the same day before the echocardiography examination. Parameters from the complete count test (neutrophil count, lymphocyte count, and hemoglobin) and biochemical test (serum creatinine, albumin, total cholesterol, and uric acid) were recorded. Transthoracic and transesophageal echocardiography were performed 24 h before the procedure. The gain was continuously adjusted until acquisition of the best image. The left ventricular ejection fraction (LVEF) was determined using Simpson’s biplane method.

### Definitions

The PNI was calculated using the following formula: 10 × serum albumin value (g/dL) + 0.005 × total lymphocyte count (per mm^3^) (8). The neutrophil-to-lymphocyte ratio (NLR) was calculated by the following equation: total neutrophil count/total lymphocyte count. The presence or absence of LAAT or SEC was determined by 2 experienced echocardiographers. Thrombus was defined as an echodense mass with uniform tissue that is different than that of the LA endocardial wall (12). SEC was defined as an echogenic, swirling pattern of blood flow at the standard gain setting during the cardiac cycle and was graded according to Fatkin classification (13). SEC graded 3 or greater was considered dense.

### Statistical Analysis

The study population was divided into three groups according to the tertiles of the PNI. Continuous variables were expressed as the mean ± standard deviation and compared among groups using ANOVA. Categorical variables were described as percentiles and compared among groups by Pearson’s chi-square or Fisher’s exact test.

The relationship between the PNI and LAAT/dense SEC was conducted using logistic analysis. The initial model was unadjusted, followed by multivariate analysis including the variables with a *P*-value < 0.1 in univariate analysis and potential risk factors for LAAT/SEC (e.g., CHA_2_DS_2_-VASc score and OAC medication). Odds ratios (ORs) and 95% confidence intervals (CIs) were calculated.

A receiver operating characteristic curve (ROC) was generated to assess the ability of the PNI to predict LAAT/dense SEC. The optimal cutoff value for the PNI was determined with the highest Youden index. All statistical analyses were performed with SPSS 19.0 (IBM, Armonk, NY, United States), and a *P*-value < 0.05 (2-tailed) was considered statistically significant.

## Results

### Baseline Characteristics

A total of 406 patients were included in this study. The baseline characteristics are displayed in Table 1. Patients with a low PNI were significantly older. The medical history and OAC medications were similar among the three groups. The neutrophil count was similar among the groups. However, the lymphocyte count was lower, resulting in a higher NLR level, in patients with a low PNI. Serum albumin, hemoglobin, and total cholesterol were greater in subjects with higher PNI tertiles. A total of 53 patients had LAAT/dense SEC detected on TEE. Patients with LAAT/dense SEC had a lower percentage of paroxysmal AF and diabetes mellitus, lower lymphocyte count, and serum albumin, greater NLR, and larger LA diameter (Supplementary Table 1).

**TABLE 1:** Baseline characteristics of the study population according to PNI.

Variables	Tertile 1 <45.5	Tertile 2 45.5–49.3	Tertile 3 >49.3	*P*-value
*N*	137	135	134	
Age, years	71.1 ± 8.9	70.0 ± 8.3	67.0 ± 8.7	<0.001
Male, *n* (%)	96 (70.1)	83 (61.5)	79 (59.0)	0.136
Body mass index, kg/m^2^	23.9 ± 3.5	24.6 ± 3.7	24.5 ± 3.2	0.221
Paroxysmal AF, *n* (%)	38 (27.7)	46 (34.1)	46 (34.3)	0.362
Hypertension, *n* (%)	88 (64.2)	91 (67.4)	92 (68.7)	0.727
Diabetes mellitus, *n* (%)	20 (14.6)	24 (17.8)	26 (19.4)	0.566
Congestive heart failure, *n* (%)	20 (14.6)	17 (12.6)	15 (11.2)	0.701
Previous TIA/stroke, *n* (%)	97 (70.8)	98 (72.6)	95 (70.9)	0.935
CHA_2_DS_2_-VASc score, points	4.6 ± 1.5	4.7 ± 1.5	4.4 ± 1.6	0.283
**Laboratory parameters**				
Neutrophil count, 10^9^/L	3.8 ± 1.4	3.8 ± 1.4	3.9 ± 1.3	0.858
Lymphocyte count, 10^9^/L	1.2 ± 0.3	1.5 ± 0.4	1.9 ± 0.5	<0.001
NLR	3.4 ± 1.6	2.6 ± 1.1	2.2 ± 1.0	<0.001
Hemoglobin, g/L	132.7 ± 18.4	136.4 ± 22.3	142.1 ± 15.5	<0.001
Serum creatinine, μmmol/L	78.9 ± 23.3	75.5 ± 18.8	73.7 ± 18.1	0.105
Serum albumin, g/L	36.8 ± 2.6	39.8 ± 2.4	42.9 ± 3.1	<0.001
Total cholesterol, mmol/L	3.5 ± 1.0	3.8 ± 1.1	4.1 ± 1.1	<0.001
Uric acid, μmmol/L	352.4 ± 96.4	353.8 ± 90.3	354.4 ± 89.7	0.983
**Echocardiograph parameters**				
LA diameter, mm	45.3 ± 7.5	45.1 ± 7.7	44.1 ± 6.8	0.346
LVEF,%	60.9 ± 7.4	61.8 ± 7.0	62.8 ± 5.3	0.064
LAA orifice diameter, mm	24.8 ± 4.8	25.0 ± 5.2	24.3 ± 4.7	0.475
LAA depth, mm	30.0 ± 5.9	28.9 ± 5.7	29.4 ± 5.5	0.255
LAAT/dense SEC, *n* (%)	28 (20.4)	19 (14.1)	6 (4.5)	<0.001
OAC medications, *n* (%)				0.738
None	20 (14.6)	17 (12.6)	21 (15.7)	
Warfarin	32 (23.4)	37 (27.4)	36 (26.9)	
Dabigatran	53 (38.7)	58 (43.0)	47 (35.1)	
Rivaroxaban	32 (23.4)	23 (17.0)	30 (22.4)	

*PNI, prognostic nutritional index; AF, atrial fibrillation; TIA, transient ischemic attack; NLR, neutrophil-to-lymphocyte ratio; LA, left atrium; LVEF, left ventricular ejection fraction; LAA, left atrial appendage, LAAT, left atrial appendage thrombus; SEC, spontaneous echo contrast; OAC, oral anticoagulation.*

### Prognostic Nutritional Index and Left Atrial Appendage Thrombus/Dense Spontaneous Echo Contrast

**FIGURE 2:**
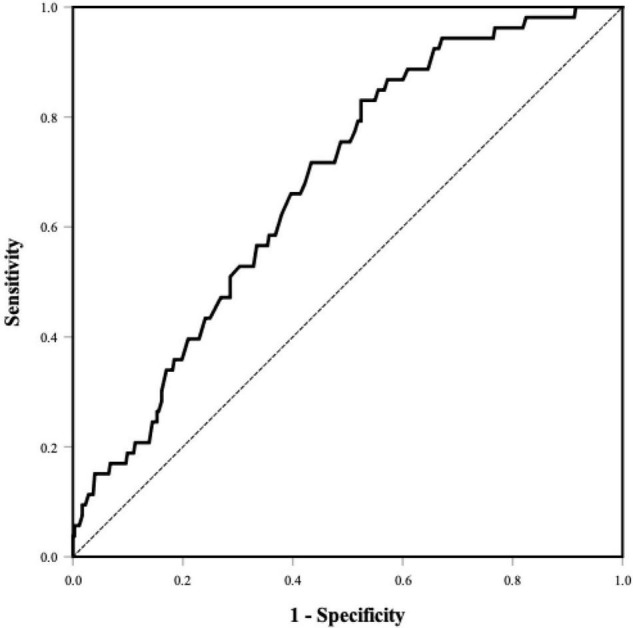
ROC curve of the PNI for predicting LAAT/dense SEC. Area under the curve was 0.68 (95% CI, 0.61–0.75, *P* < 0.001). The optional cutoff value of the PNI was 48.0 (sensitivity 83.0%, specificity 47.6%). PNI, prognostic nutritional index; LAAT, left atrial appendage thrombus; SEC, spontaneous echo contrast.

The percentages of LAAT/dense SEC were 20.4, 14.1, and 4.5% in PNI tertiles 1–3, respectively (Table 1). The PNI was lower in the individuals with LAAT/dense SEC (45.0 ± 4.0 vs. 47.9 ± 4.5; *P* < 0.001; Supplementary Table 1). A lower PNI was associated with a higher prevalence of LAAT/dense SEC in the univariate logistic regression analysis. In the multivariate analysis model, the PNI was an independent predictor for LAAT/dense SEC (OR 0.89; 95% CI, 0.82–0.97; *P* = 0.007; Table 2). In addition, elevated NLR showed a positive relationship with increased LAAT/dense SEC (Table 2). ROC analysis revealed that the optimal cutoff value of the PNI for predicting LAAT/dense SEC was 48.0 (area under the curve: 0.68; 95% CI, 0.61–0.75; *P* < 0.001; Figure 2). The sensitivity and specificity were 83.0 and 47.6%, respectively. Multivariate logistic regression showed that a PNI ≤ 48.0 was independently associated with LAAT/dense SEC (OR 3.57; 95% CI, 1.41–9.02; *P* = 0.007; Table 2).

**TABLE 2:** Univariate and multivariate logistic regression models to identify the in predictors of LAAT/dense SEC.

Variables	Univariate model		Multivariate model 1^a^		Multivariate model 2^b^	
			
	OR (95% CI)	*P*-value	OR (95% CI)	*P*-value	OR (95% CI)	*P*-value
Age	0.99 (0.96–1.03)	0.730	0.97 (0.93-1.01)	0.134	0.97 (0.93-1.01)	0.134
Male	1.03 (0.57–1.88)	0.922	0.70 (0.34–1.43)	0.327	0.73 (0.36–1.50)	0.395
Body mass index	1.00 (0.92–1.08)	0.943	−	−	−	−
Paroxysmal AF	0.34 (0.15–0.74)	0.006	0.51 (0.21–1.20)	0.123	0.48 (0.20–1.13)	0.093
Hypertension	1.31 (0.69–2.47)	0.413	−	−	−	−
Diabetes mellitus	0.36 (0.12–1.02)	0.054	0.49 (0.16–1.51)	0.486	0.44 (0.14–1.40)	0.165
Congestive heart failure	1.48 (0.67–3.23)	0.332	−	−	−	−
Previous TIA/stroke	0.68 (0.37–1.25)	0.210	−	−	−	−
CHA_2_DS_2_-VASc score	0.90 (0.74–1.09)	0.273	1.00 (0.76–1.31)	0.998	1.00 (0.76–1.32)	0.987
NLR	1.75 (1.41–2.18)	<0.001	1.65 (1.29–2.11)	<0.001	1.66 (1.30–2.11)	<0.001
Hemoglobin	1.01 (0.995–1.03)	0.165	−	−	−	−
Serum creatinine	1.01 (0.99–1.02)	0.431	−	−	−	−
Total cholesterol	1.03 (0.79–1.34)	0.856	−	−	−	−
LA diameter	1.06 (1.02–1.10)	0.005	1.04 (0.995–1.09)	0.079	1.04 (0.99–1.09)	0.094
LVEF	0.98 (0.94–1.02)	0.232	−	−	−	−
LAA orifice diameter	1.01 (0.95–1.07)	0.876	−	−	−	−
LAA depth	1.04 (0.99–1.09)	0.152	−	−	−	−
OAC medication	1.35 (0.55–3.33)	0.510	1.78 (0.66–4.81)	0.257	1.79 (0.65–4.90)	0.257
PNI	0.86 (0.80–0.92)	<0.001	0.89 (0.82–0.97)	0.007	−	−
PNI ≤ 48.0	4.97 (2.07–11.93)	<0.001	−	−	3.57 (1.41-9.02)	0.007

*OR, odds ratio; CI, confidence interval; PNI, prognostic nutritional index; LAAT, left atrial appendage thrombus; SEC, spontaneous echo contrast; AF, atrial fibrillation; TIA, transient ischemic attack; NLR, neutrophil-to-lymphocyte ratio; LA, left atrium; LVEF, left ventricular ejection fraction; LAA, left atrial appendage; OAC, oral anticoagulation; PNI, prognostic nutritional index.*

*^a^Multivariate model 1: including age, sex, paroxysmal AF, DM, LA diameter, CHA_2_DS_2_-VASc score, NLR, OAC medication, and PNI.*

*^b^Multivariate model 2: including age, sex, paroxysmal AF, DM, LA diameter, CHA_2_DS_2_-VASc score, NLR, OAC medication, and PNI ≤ 48.0.*

## Discussion

In the present study, lymphocyte count and serum albumin were both lower in patients with LAAT/dense SEC. Multivariate logistic regression analysis showed that the PNI was an independent predictor for LAAT/dense SEC. The risk of LAAT/dense SEC in patients with a PNI ≤ 48.0 was 2.57-fold higher than that in those with a PNI > 48.0.

The PNI was initially used as a prognostic index in patients with cancer (14, 15). A lower PNI was associated with a higher mortality risk. The PNI was then applied to cardiovascular diseases, including acute myocardial infarction (8), acute heart failure (7), and atrial fibrillation (10). All studies indicated that the PNI was an independent predictor of clinical outcomes, and the predictive power of the PNI was higher than that of albumin or lymphocyte count alone. Recently, Engin et al. reported that a low PNI was a risk factor for postoperative AF in patients undergoing coronary artery bypass grafting (10). Furui et al. found that the AF recurrence rate after catheter ablation was higher in patients with undernutrition than in those with normal nutrition stratified by nutrition scoring tools (11).

Paar et al. examined the influence of albumin on blood coagulation *in vitro*. The changes in hemostatic profiling indicated increased primary hemostasis, improved platelet aggregation, and enhanced clot formation in the low albumin group. Therefore, they claimed that low albumin levels might contribute to frequently occurring venous thromboembolism (16). Pennington et al. reported that lower serum albumin was an independent predictor for venous thromboembolism in patients with spinal tumors (17). In a prospective cohort study, decreased serum albumin was associated with an increased risk of venous thromboembolism (18). Therefore, low serum albumin may be a marker of a hypercoagulable state. However, the role of nutritional markers in the LAA thrombogenic milieu is still unknown.

To our knowledge, this study was the first to investigate the association of the PNI with LAAT/dense SEC in patients with AF. We found that the PNI was inversely related to LAAT/dense SEC. The PNI is a composite indicator of inflammation (lymphocytes) and nutritional status (serum albumin). Therefore, the findings of the current study may be explained as follows. First, patients with a low PNI demonstrated malnutrition. We also found that hemoglobin and total cholesterol levels were significantly lower in subjects with a low PNI. Once LAAT is detected, OACs should be prescribed for at least 3 weeks, and repeated transesophageal echocardiography should be performed (19). Both warfarin and NOACs have been proven to be effective in the treatment of LAAT (20). However, in patients with nutritional problems, food interaction may influence the efficacy of OACs (especially warfarin) for thromboembolic prevention (21). A recent investigation demonstrated that OAC changing was associated with LA/LAA thrombus resolution in more than 50% of cases, especially after a switch to a new full-dose NOAC (22). Therefore, direct oral anticoagulants may be preferred to warfarin due to fewer food interactions (23). Second, inflammation may play a role in the thrombogenic milieu in the present study. Although neutrophil count was comparable between patients with high and low PNI values, it was significantly higher in patients with LAAT/dense SEC than in those without LAAT/dense SEC. INR, an inflammatory marker, was greater in patients with either increased PNI values or LAAT/dense SEC. Previous studies demonstrated that there was an apparent link between thrombogenesis and inflammation (24, 25).

There is currently no evidence that induction of elevated serum albumin levels (such as albumin infusion, nutrition improvement, and protein supplementation, etc.) reduces the risk of LAAT/dense SEC. However, the measurements of serum albumin and lymphocyte count are recommended to be involved in routine follow-up to investigate the trend of the PNI and the clinical benefit of whether serum albumin plays a role in the prevention of LAAT/dense SEC. It should be noted that a low PNI, indicating decreased serum albumin and lymphocyte count levels, implies a poor nutritional state, which usually leads to poor clinical outcomes.

There were several limitations in the current study. First, this was a single-center, retrospective, and observational study. Therefore, the exact causal relationship between the PNI and LAAT/dense SEC is unknown. Second, although we used multivariable analysis, we could not exclude the possibility of residual unmeasured covariables that might influence the outcomes. Third, patients in the current study were candidates for percutaneous LAA closure with relatively high stroke and/or bleeding risk. Whether these results can be extended to all AF patients is unknown.

## Conclusion

This study was the first to investigate the association between the PNI and LAAT/dense SEC in patients with non-valvular AF. The results indicated that the PNI was inversely correlated with LAAT/dense SEC. Therefore, it may be considered a predictor for LAAT/dense SEC. Further prospective studies with a larger number of patients are required to validate our findings.

## Data Availability Statement

The raw data supporting the conclusions of this article will be made available by the authors, without undue reservation.

## Ethics Statement

The studies involving human participants were reviewed and approved by Ethics Committee of Ningbo First Hospital. The patients/participants provided their written informed consent to participate in this study. Written informed consent was obtained from the individual(s) for the publication of any potentially identifiable images or data included in this article.

## Author Contributions

ZW and BW designed the study and drafted the manuscript and did the statistical analysis. ZW, BW, and GF collected patient data and edited the images. BH, HC, and SZ critically revised the manuscript and approved the article. All authors contributed to the article and approved the submitted version.

## Conflict of Interest

The authors declare that the research was conducted in the absence of any commercial or financial relationships that could be construed as a potential conflict of interest.

## Publisher’s Note

All claims expressed in this article are solely those of the authors and do not necessarily represent those of their affiliated organizations, or those of the publisher, the editors and the reviewers. Any product that may be evaluated in this article, or claim that may be made by its manufacturer, is not guaranteed or endorsed by the publisher.

## References

[1] Sheikh A Patel NJ Nalluri N Agnihotri K Spagnola J Patel A Trends in hospitalization for atrial fibrillation: epidemiology, cost, and implications for the future. *Prog Cardiovasc Dis.* (2015) 58:105–16. 10.1016/j.pcad.2015.07.002 26162957

[2] Wolf PA Abbott RD Kannel WB. Atrial fibrillation as an independent risk factor for stroke: the Framingham study. *Stroke.* (1991) 22:983–8. 10.1161/01.str.22.8.983 1866765

[3] Al-Saady NM Obel OA Camm AJ. Left atrial appendage: structure, function, and role in thromboembolism. *Heart.* (1999) 82:547–54. 10.1136/hrt.82.5.547 10525506 PMC1760793

[4] Black IW Chesterman CN Hopkins AP Lee LC Chong BH Walsh WF. Hematologic correlates of left atrial spontaneous echo contrast and thromboembolism in nonvalvular atrial fibrillation. *J Am Coll Cardiol.* (1993) 21:451–7. 10.1016/0735-1097(93)90688-w 8426010

[5] Gedikli O Mohanty S Trivedi C Gianni C Chen Q Della Rocca DG Impact of dense “smoke” detected on transesophageal echocardiography on stroke risk in patients with atrial fibrillation undergoing catheter ablation. *Heart Rhythm.* (2019) 16:351–7. 10.1016/j.hrthm.2018.10.004 30312757

[6] Wang B Wang Z Fu G He B Wang H Zhuo W Left atrial spontaneous echo contrast and ischemic stroke in patients undergoing percutaneous left atrial appendage closure. *Front Cardiovasc Med.* (2021) 8:723280. 10.3389/fcvm.2021.723280 34631825 PMC8495018

[7] Cheng YL Sung SH Cheng HM Hsu PF Guo CY Yu WC Prognostic nutritional index and the risk of mortality in patients with acute heart failure. *J Am Heart Assoc.* (2017) 6:e004876. 10.1161/JAHA.116.004876 28649089 PMC5669149

[8] Keskin M Hayiroglu MI Keskin T Kaya A Tatlisu MA Altay S A novel and useful predictive indicator of prognosis in ST-segment elevation myocardial infarction, the prognostic nutritional index. *Nutr Metab Cardiovasc Dis.* (2017) 27:438–46. 10.1016/j.numecd.2017.01.005 28392077

[9] Wang Z Zhao L He S. Prognostic nutritional index and the risk of mortality in patients with hypertrophic cardiomyopathy. *Int J Cardiol.* (2021) 331:152–7. 10.1016/j.ijcard.2021.01.023 33529655

[10] Engin M Ozsin KK Savran M Guvenc O Ozyazicioglu AF. Visceral adiposity index and prognostic nutritional index in predicting atrial fibrillation after on-pump coronary artery bypass operations: a prospective study. *Braz J Cardiovasc Surg.* (2020) 36:522–9. 10.21470/1678-9741-2020-0044 33355787 PMC8522311

[11] Furui K Morishima I Morita Y Kanzaki Y Takagi K Nagai H Impact of preoperative nutritional status on the outcome of catheter ablation for atrial fibrillation. *Circ J.* (2021) 86:268–76. 10.1253/circj.CJ-21-0218 34373432

[12] Aschenberg W Schluter M Kremer P Schroder E Siglow V Bleifeld W. Transesophageal two-dimensional echocardiography for the detection of left atrial appendage thrombus. *J Am Coll Cardiol.* (1986) 7:163–6. 10.1016/s0735-1097(86)80275-3 3941205

[13] Fatkin D Loupas T Jacobs N Feneley MP. Quantification of blood echogenicity: evaluation of a semiquantitative method of grading spontaneous echo contrast. *Ultrasound Med Biol.* (1995) 21:1191–8. 10.1016/0301-5629(95)02006-3 8849833

[14] Pinato DJ North BV Sharma R. A novel, externally validated inflammation-based prognostic algorithm in hepatocellular carcinoma: the prognostic nutritional index (PNI). *Br J Cancer.* (2012) 106:1439–45. 10.1038/bjc.2012.92 22433965 PMC3326674

[15] Zhang W Ye B Liang W Ren Y. Preoperative prognostic nutritional index is a powerful predictor of prognosis in patients with stage III ovarian cancer. *Sci Rep.* (2017) 7:9548. 10.1038/s41598-017-10328-8 28842710 PMC5573316

[16] Paar M Rossmann C Nusshold C Wagner T Schlagenhauf A Leschnik B Anticoagulant action of low, physiologic, and high albumin levels in whole blood. *PLoS One.* (2017) 12:e0182997. 10.1371/journal.pone.0182997 28800610 PMC5553770

[17] Pennington Z Ehresman J Schilling A Feghali J Hersh AM Hung B Influence of tranexamic acid use on venous thromboembolism risk in patients undergoing surgery for spine tumors. *J Neurosurg Spine.* (2021) 35:663–673. 10.3171/2021.1.SPINE201935 34388705

[18] Kunutsor SK Seidu S Katechia DT Laukkanen JA. Inverse association between serum albumin and future risk of venous thromboembolism: interrelationship with high sensitivity C-reactive protein. *Ann Med.* (2018) 50:240–8. 10.1080/07853890.2018.1441537 29448840

[19] Hindricks G Potpara T Dagres N Arbelo E Bax JJ Blomstrom-Lundqvist C 2020 ESC guidelines for the diagnosis and management of atrial fibrillation developed in collaboration with the European association for cardio-thoracic surgery (EACTS). *Eur Heart J.* (2021) 42:373–498. 10.1093/eurheartj/ehaa612 32860505

[20] Murtaza G Turagam MK Atti V Garg J Boda U Velagapudi P Warfarin vs non-vitamin K oral anticoagulants for left atrial appendage thrombus: a meta-analysis. *J Cardiovasc Electrophysiol.* (2020) 31:1822–7. 10.1111/jce.14502 32323386

[21] Granziera S Cohen AT Nante G Manzato E Sergi G. Thromboembolic prevention in frail elderly patients with atrial fibrillation: a practical algorithm. *J Am Med Dir Assoc.* (2015) 16:358–64. 10.1016/j.jamda.2014.12.008 25680239

[22] Di Cori A Barletta V Meola L Parollo M Mazzocchetti L Carluccio M Left atrial thrombus and smoke resolution in patients with atrial fibrillation under chronic oral anticoagulation. *J Interv Card Electrophysiol.* (2022). 10.1007/s10840-022-01169-1 [Epub ahead of print]. 35277775

[23] Zhu J Gao RJ Liu Q Jiang RH Yu L Sun YX Metabolic benefits of rivaroxaban in non-valvular atrial fibrillation patients after radiofrequency catheter ablation. *J Zhejiang Univ Sci B.* (2017) 18:946–54. 10.1631/jzus.B1600492 29119732 PMC5696313

[24] Joseph L Fink LM Hauer-Jensen M. Cytokines in coagulation and thrombosis: a preclinical and clinical review. *Blood Coagul Fibrinolysis.* (2002) 13:105–16. 10.1097/00001721-200203000-00005 11914652

[25] Libby P Simon DI. Inflammation and thrombosis: the clot thickens. *Circulation.* (2001) 103:1718–20. 10.1161/01.cir.103.13.1718 11282900

