# Supplementary material for: The Prognostic Nutritional Index May Predict Left Atrial Appendage Thrombus or Dense Spontaneous Echo Contrast in Patients With Atrial Fibrillation
Source: Front Cardiovasc Med. 2022 Apr 29;9:860624. doi: 10.3389/fcvm.2022.860624 (PMC9098831; doi:10.3389/fcvm.2022.860624)
Supplement: Supplementary file 1 [file Table_1.DOCX]

Supplementary Material

**Supplementary Table 1** Baseline characteristics of the patients with and without LAAT/dense SEC

| Variables | LAAT/dense SEC (+) | LAAT/dense SEC (−) | P value |
| --- | --- | --- | --- |
| n | 53 | 353 |  |
| Age, years | 69.0±9.5 | 69.4±8.7 | 0.731 |
| Male, n (%) | 34 (64.2) | 224 (63.5) | 0.922 |
| Body mass index, kg/m^2^ | 24.3±3.5 | 24.4±3.4 | 0.943 |
| Paroxysmal AF, n (%) | 8 (15.1) | 122 (34.6) | 0.005 |
| Hypertension, n (%) | 38 (71.7) | 233 (66.0) | 0.412 |
| Diabetes mellitus, n (%) | 4 (7.5) | 66 (18.7) | 0.045 |
| Congestive heart failure, n (%) | 9 (17.0) | 43 (12.2) | 0.330 |
| Previous TIA/stroke, n (%) | 34 (64.2) | 256 (72.3) | 0.208 |
| CHA_2_DS_2_-VASc score, points | 4.4±1.7 | 4.6±1.5 | 0.273 |
| Laboratory parameters |  |  |  |
| Neutrophil count, 10^9^/L | 4.5±1.8 | 3.7±1.3 | 0.004 |
| Lymphocyte count, 10^9^/L | 1.3±0.3 | 1.6±0.5 | <0.001 |
| NLR | 3.8±1.9 | 2.6±1.2 | <0.001 |
| Hemoglobin, g/L | 140.5±18.9 | 136.5±19.3 | 0.167 |
| Serum creatinine, µmmol/L | 78.1±22.8 | 75.8±19.9 | 0.432 |
| Serum albumin, g/dL | 39.0±3.3 | 40.1±3.4 | 0.036 |
| PNI | 45.0±4.0 | 47.9±4.5 | <0.001 |
| Total cholesterol, mmol/L | 3.8±1.2 | 3.8±1.1 | 0.856 |
| Uric acid, µmmol/L | 374.1±95.4 | 350.4±91.2 | 0.402 |
| Echocardiograph parameters |  |  |  |
| LA diameter, mm | 47.6±6.6 | 44.5±7.4 | 0.004 |
| LVEF, % | 60.8±6.3 | 62.0±6.7 | 0.231 |
| LAA orifice diameter, mm | 24.8±4.4 | 24.7±5.0 | 0.876 |
| LAA depth, mm | 30.5±4.9 | 29.3±5.8 | 0.151 |
| OAC medications, n (%) |  |  | 0.497 |
| None | 6 (11.3) | 52 (14.7) |  |
| Warfarin | 17 (32.1) | 88 (24.9) |  |
| Dabigatran | 22 (41.5) | 136 (38.5) |  |
| Rivaroxaban | 8 (15.1) | 77 (21.8) |  |

PNI, prognostic nutritional index; LAAT, left atrial appendage thrombus; SEC, spontaneous echo contrast; AF, atrial fibrillation; TIA, transient ischemic attack; NLR, neutrophil-to-lymphocyte ratio; LA, left atrium; LVEF, left ventricular ejection fraction; LAA, left atrial appendage; OAC, oral anticoagulation.
